# Autism and Down syndrome: early identification and diagnosis

**DOI:** 10.1590/0004-282X-ANP-2021-0156

**Published:** 2022-08-08

**Authors:** Natália Lisce Fioravante Diniz, Erika Parlato-Oliveira, Priscila Gonçalves Ayres Pimenta, Liubiana Arantes de Araújo, Eugênia Ribeiro Valadares

**Affiliations:** 1 Universidade Federal de Minas Gerais, Faculdade de Medicina, Programa Saúde da Criança e do Adolescente, Belo Horizonte MG, Brasil.; 2 CRPMS, UFR Études Psychanalytiques, Université Paris Diderot, Paris, France; 3 CRPMS, Université de Paris, Paris, France.; 4 Instituto Langage, São Paulo, Brasil.

**Keywords:** Autism Spectrum Disorder, Down Syndrome, Diagnosis, Transtorno do Espectro Autista, Síndrome de Down, Diagnóstico

## Abstract

**Background:**

The diagnosis of autism spectrum disorder (ASD) in Down syndrome (DS) is underestimated because it is necessary to understand which aspects of the behavioral phenotype are related to DS and which are related to ASD.

**Objective:**

To conduct a systematic review of the literature on early identification and diagnosis of ASD in patients with DS.

**Data source:**

The VHL, MEDLINE, Cochrane, CINAHL, Scopus, Web of Science and Embase databases were searched and data were evaluated using PRISMA.

**Data synthesis:**

Out of 1,729 articles evaluated, 15 were selected. Although well studied, identification of ASD in DS can be difficult because of the need to understand which aspects of the behavioral phenotype are related to Down syndrome and which to autism. In this review, the prevalence of ASD was found to range from 12% to 41%. Early identification of autism risk in individuals with Down syndrome is still poorly studied, even though there are screening instruments for infants. Several instruments for diagnosing autism in individuals with Down syndrome were found, but a developmental approach is fundamental for making a clear diagnosis

**Conclusions:**

Screening procedures are important for detecting early signs of autism risk in the first year of life. Careful evaluation methods are needed to establish the diagnosis, which include choosing appropriate tools for evaluation of development and cognition, and analysis of qualitative aspects of social interaction, among others. It has been indicated in the literature that early detection and timely accurate diagnosis, in association with an intervention, may benefit development, quality of life and social inclusion.

## RESUMO

**Antecedentes:**

O diagnóstico de autismo na síndrome de Down é subestimado, sendo necessário entender quais aspectos do fenótipo comportamental estão relacionados à síndrome de Down e quais são do autismo.

**Objetivo:**

Revisão Sistemática da Literatura sobre identificação precoce e diagnóstico do Transtorno do Espectro Autista em pacientes com síndrome de Down. Fonte de dados: Busca nas bases BVS, MEDLINE, Cochrane, CINAHL, Scopus, Web of Science e Embase e avaliação pelo PRISMA.

**Síntese dos dados:**

De 1.729 artigos avaliados, foram selecionados 15. Apesar de ser bastante estudada, a identificação do transtorno do espectro do autismo na síndrome de Down pode ser difícil devido a compreensão de quais aspectos do fenótipo comportamental estão relacionados à síndrome de Down e quais são do autismo. Nessa revisão foi encontrada variação na prevalência de 12% a 41%. A identificação precoce de risco de autismo na síndrome de Down é pouco estudada mesmo existindo instrumentos de triagem para lactentes. Sobre o diagnóstico do autismo na síndrome de Down foram encontrados diversos instrumentos, mas é necessária abordagem desenvolvimental para um diagnóstico apurado.

**Conclusões:**

É destacada a importância de procedimentos de triagem de sinais precoces de risco de autismo ainda no primeiro ano de vida. São para estabelecimento do diagnóstico a escolha de instrumentos para a avaliação do desenvolvimento e cognição, análise dos aspectos qualitativos da interação social, dentre outros. A detecção precoce e o diagnóstico preciso no tempo correto e uma intervenção poderão beneficiar o desenvolvimento, a qualidade de vida e inclusão social.

## INTRODUCTION

Down Syndrome (DS) is a common chromosomal anomaly and affects around 1 in 1000 individuals^1^ ^2^. Recent research has indicated that the prevalence of autism spectrum disorder (ASD) is higher among individuals with DS^1^. 

ASD consists of a heterogeneous group of neurodevelopmental disorders that are characterized by disorders of social relations and communication, repetitive behaviors and restricted interests^3^. According to data from the CDC, this disorder affects approximately 1 in 54 individuals^4^. Another study showed that the ASD rate was 1 in 100 children born in the United States^5^. Data on the prevalence of ASD in DS vary, since studies have indicated that ASD affects between 2% and 10% of the population with DS, a rate that is higher than in the general population^2^ ^6^. 

The diagnosis of ASD in Down syndrome is underestimated, because it is necessary to understand which aspects of the behavioral phenotype are related to DS and which are related to ASD^7^.

Several standardized scales have been used to evaluate the ASD criteria^8^ ^9^. Some researchers have recommended a developmental approach to the diagnosis of ASD among cognitively impaired children, i.e. the social or communication function needs to be qualitatively different and more impaired than the general cognitive function, for an additional ASD diagnosis to be made. Researchers taking this approach have generally reported lower ASD prevalence. Epidemiological studies using a developmental approach to estimate the prevalence of autism among children with Down syndrome are scarce^8^ ^10^. 

The discussion about which criteria and instruments should be used to diagnose ASD in DS has been the subject of some studies, because the tools generally used have been validated considering individuals who do not have specific syndromes but who do have different levels of development. Furthermore, these tools do not exclude individuals with functional disorders, such as are present in DS^2^.

In such cases, professionals should consider whether individuals’ communicative social functioning corresponds to their basal level of development. In the absence of a developmental perspective, delays that are symptoms of a social disorder, e.g. ASD, can be misinterpreted^1^ ^2^. 

Another aspect of ASD is that, when early signs of risk of this disorder are identified and the intervention occurs in the first year of life, the chances of successful therapies are greater^11^ ^-^ ^13^. Intervention at earlier ages is favored because this is the time of greatest potential for neural plasticity. Studies on detection and intervention mechanisms are increasingly necessary^14^ ^15^. Identification of early signs of autism risk (before one year of age) occurs at a developmental point at which the diagnosis is more difficult to make. Intervention at this point will aim to modify the trajectory and change the prognosis^16^. 

Identification of early signs of autism risk has been widely studied, since no biomarker for the diagnosis of autism currently exists. The diagnosis is still made late, at around three years of age, even though symptoms are present in the first years of life^17^. In cases of DS, the diagnosis tends to be made even later^18^.

With increasing numbers of studies on the early signs of autism risk, screening tools such as M-CHAT R have been created and tested at younger ages. In Brazil, law 13.438 recommends that formal evaluation of child development should be conducted on all infants using the *Caderneta da Criança* (Children's Booklet), which contains data that can guide ASD screening. Nonetheless, even with the increase in research, diagnostic tools for children, such as CARS, ADI-R and ADOS, are mainly concentrated around the age of two years. There are also tools that evaluate children in the first year, but few before the first year of life^17^.

Identification of early signs of autism risk may lead to interventions at the most appropriate time and with better results^17^. Children with DS often present considerable delay in receiving the diagnosis of ASD, and this may result in inadequate strategies^13^ ^11^. Attending to the need for earlier interventions, tools have been used to detect signs of autism in the first months of the child's life^17^. 

These interventions can prevent or minimize autism symptoms, such as premature appearance of stereotypes, isolation and communication delay. These are the symptoms that can subsequently lead to a diagnosis of ASD, particularly among children with DS^19^.

In this regard, understanding how to identify early signs of autism risk among infants and make the diagnosis of autism in the population of people with DS is important, given the propositions that are necessary in these contexts. Faced with this issue, we conducted a systematic review on ASD in DS.

## METHODS

This was a systematic review of the literature based on PRISMA (Preferred Reporting Items for Systematic Reviews and Meta‐Analyses)^20^. A search was conducted in the BVS, MEDLINE, Cochrane, CINAHL, Scopus, Web of Science and Embase databases to identify the main studies that evaluated autism spectrum disorder in Down syndrome.

### Search strategy

To search for articles, specific descriptors linked to Boolean operators (AND and OR) were used with the aid of parentheses - ( ) - to delimit intercalations within the same logic and quotation marks (") to identify compound words. Therefore, the descriptors were applied as follows: "Autistic Disorder" OR "Trastorno do Espectro do Autismo" OR "Transtorno do Espectro Autista" OR autismo OR "Autismo Infantil" OR "Síndrome de Kanner" OR autism OR "Autism, Early Infantile" OR "Disorder, Autistic" OR "Disorders, Autistic" OR "Early Infantile Autism" OR "Infantile Autism" OR "Infantile Autism, Early" OR "Kanner Syndrome" OR "Kanners Syndrome" OR "Autism, Infantile" OR "Kanner's Syndrome") AND (tw: "Down Syndrome" OR "Síndrome de Down" OR "Síndrome de Down" OR "Down Syndrome, Partial Trisomy 21" OR "Down's Syndrome" OR "Partial Trisomy 21 Down Syndrome" OR "Downs Syndrome" OR "Syndrome, Down" OR "Syndrome, Down's”. This search was conducted in June and July 2019.

No filters such as article language, target audience or publication deadline were added. No such limitation were imposed because the objective was to include the largest number of articles relating to the prevalence of ASD in DS.

### Recruitment and selection bias

To select potentially eligible articles, after exporting the studies selected from the databases, the Rayyan software was used. This is specific software for systematic reviews, in the form of a web and mobile app  ^21^. After importing the search results, the following steps were conducted: a) identification ‐ recruitment of studies; b) selection ‐ exclusion of duplicates and exclusion through reading of titles and abstracts; c) eligibility ‐ exclusion through full reading of the studies; and d) inclusion ‐ eligible studies, according to pre-established inclusion criteria. 

The whole process was carried out by two independent researchers and was assessed by a third reviewer by reading the titles and abstracts. It should be noted that two inclusion or exclusion criteria were followed: a) articles selected by both researchers were included; and b) articles selected by only one researcher were analyzed by the third reviewer and, if these fitted the criteria, they were included. A further search was performed through reading the reference lists of the studies included in the eligibility phase (full reading of the articles).

### Inclusion criteria

The criteria for inclusion of articles were the following: a) eligible cross-sectional epidemiological studies describing the prevalence of autism in the population with Down syndrome; b) eligible studies that presented the specificities of the diagnosis of autism in Down syndrome, with a detailed approach to diagnostic methods; c) eligible studies that showed the identification of signs of autism risk in the population of infants with Down syndrome; and d) no restrictions regarding age, gender, class of healthcare professional or the date of use of the service. Studies in English and Spanish were included.

Studies that did not demonstrate the criteria for the diagnosis of autism and those conducted prior to 2000 were excluded.

### Data extraction

The data from each article were distributed in a table. The following information was included: country, year of publication, study design and data collection tools.

The quality of the evidence was evaluated in accordance with the criteria proposed by the EPHPP (Effective Public Health Practice Project - Quality Assessment Toll for Quantitative Studies - Annex 3)^22^These criteria evaluate the selection bias, study design, potential confounding factors, blinding of the investigator and participant, method of data collection, loss of follow-up, integrity of the intervention and appropriate analysis of the research question. Based on these criteria, studies were then classified as having weak, moderate or strong quality of evidence.

## RESULTS

**Figure 1.**
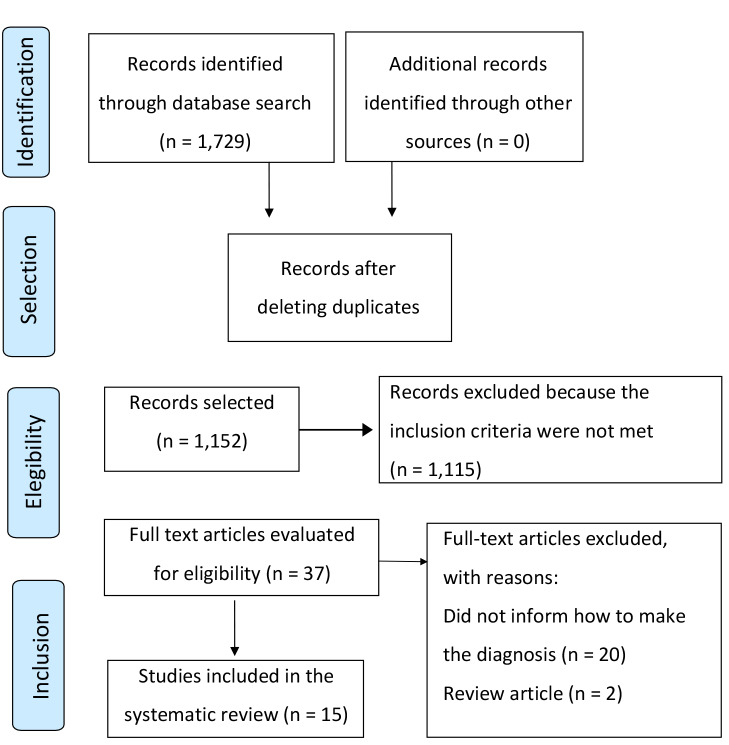
Flow of studies included in the review - PRISMA (Preferred Reporting Items for Systematic Reviews and Meta‐Analyses).

The search based on the proposed content resulted in retrieval of 1,729 articles. Out of these, 577 duplicates were excluded, and 1,149 articles were selected for reading the titles and abstracts. Through this first analysis, 37 articles were selected for full reading. Out of these, 15 articles met the inclusion criteria for review. Figure 1 shows the selection flowchart for the studies. Table 1 shows the general characteristics of the articles included in this review.

**Table 1.** General characteristics of the studies included in the systematic review^*^.

Author, year	Location	Study design	Origin and sample size	Age range	Study outcome	Quality of evidence
Ortiz et al.^22^, 2017	Catalonia, Spain	Retrospective cohort	Down's Medical Centre (12)	0 to 5 years	Identification of early signs of ASD in DS	Weak
Starr et al.^23^, 2005	Manchester, Liverpool and Leeds, England	Cohort	Down Syndrome Association, UK (13)	7 to 31 years	Diagnosis of ASD in DS	Weak
Carter et al.^24^, 2006	USA	Cohort	Not informed (127)	2 to 24 years	Diagnosis of ASD in DS	Weak
Hepburn et al.^6^, 2007	Denver, USA	Cohort	Mile High Down Syndrome Association (20)	2 to 3 years	Diagnosis of ASD in DS	Weak
Dressler et al.^18^, 2011	Pisa, Livorno, Bologna and Pistoia, Italy	Cohort	IRCCS Stella Maris Foundation, University of Pisa (24)	6 to 34 years	Diagnosis of ASD in DS	Weak
Ji et al.^25^, 2011	USA	Cohort	Kennedy Krieger Institute Down Syndrome Clinic (293)	2 to 13 years	Diagnosis of ASD in DS	Weak
Magyar et al.^26^, 2012	USA	Cohort	Participants in a prevalence study (71)	4 to 14 years	Diagnosis of ASD in DS	Weak
Pandolfi et al.^7^, 2017	USA	Cohort	Participants in a prevalence study (71)	3 to 15 years	Diagnosis of ASD in DS	Weak
Godfrey et al.^27^, 2019	USA	Cohort	Parents’ association (18)	Born between 1996 and 2003	Diagnosis of ASD in DS	Weak
Oxelgren et al.^28^, 2019	Uppsala, Sweden	Cohort	Uppsala University Children's Hospital (60)	5 to 17 years	Diagnosis of ASD in DS	Weak
Capone et al.^29^, 2005	Baltimore, USA	Cohort	Kennedy Krieger Institute (131)	2 to 21 years	Diagnosis of ASD in DS	Weak
DiGuiseppi et al.^8^, 2010	Colorado, USA	Cross-sectional	General population and the Mile High Down Syndrome Association (123)	2 to 11 years	Diagnosis of ASD in DS	Weak
Moss et al.^30^, 2013	Birmingham and London, UK	Cross-sectional	Down Syndrome Association, UK (108)	4 to 62 years	Diagnosis of ASD in DS	Weak
Warner et al.^31^, 2014	England and Wales	Cohort	Down Syndrome Association, UK (160)	4 to 40 years	Diagnosis of ASD in DS	Weak

Out of the studies included in the systematic review, 13 were cohort studies^7^ ^18^ ^23^ ^-^ ^28^ ^30^ ^32^, among which one was retrospective^23^, and two were cross-sectional^29^ ^33^These studies were published between 2005 and 2019. The ages of the subjects ranged from two to 40 years and the sample sizes ranged from 12 to 293 people. Seven studies were conducted in European countries and eight in the United States. Regarding outcomes, only one study identified early signs of autism risk^22^In addition, nine diagnosed autism^6^ ^7^ ^18^ ^24^ ^-^ ^29^ and five, the diagnosis and prevalence of ASD in DS^8^ ^28^ ^30-32^The quality of the evidence was evaluated in accordance with the criteria proposed by the EPHPP and the articles were classified as having weak quality of evidence. 

Among these fifteen studies included, the use of screening assessment tools and autism diagnosis varied. In the study by Ortiz et al.^22^which was the only one that aimed to identify early signs of autism risk (Table 2), we opted to use a tool based on other standardized ones, although there are mechanisms for this purpose that have already been validated. In the fourteen studies (Table 3) that presented the diagnosis of ASD as an outcome, only Capone et al.^29^did not show any use of tools validated for evaluation. Also, in relation to the diagnosis, the reference criterion varied. Six studies^24^ ^-^ ^28^ ^30^ ^33^used the DSM-IV as the reference and four^6^ ^-^ ^8^ ^18^used the DSM-IV TR. One^24^additionally used the ICD-10 and another^26^additionally used the DSM-IIIR. Two studies^28^ ^32^used the DSM-V. Three studies^28^ ^29^ ^31^did not report the diagnostic criterion. 

**Table 2:** Methodological characteristics of the studies, for identifying early signs of autism risk.

Author, year	Study design	Inclusion criteria	Sample size	Age range (years)	Autism assessment tools	Information source	Proportions of men/women
Ortiz et al.^22^, 2017	Retrospective cohort	Individuals with DS and with SD and autism	Down's Medical Centre (12)	0 to 5	Modified Checklist for Autism in Toddlers (M-CHAT) and Autism Diagnostic Interview-Revised (ADI-R)	Analysis of home videos	2:1

**Table 3.** Methodological characteristics of studies on the diagnosis and prevalence of autism in DS.

Author, year	Inclusion criteria	Sample size	Age range (years)	Autism assessment tools	Information sources	Reference criterion for diagnosis	Diagnostic benchmark prevalence	Proportions of men/women
Starr et al.^23^, 2005	Individuals with DS and severe intellectual disability	13	7 to 31	Autism Diagnostic Interview-Revised (ADI-R) and Adapted Pre-Linguistic Autism Diagnostic Observation Schedule (A-PL-ADOS)	Parents	DSM-IV and CID-10	-	1.16:1
Carter et al.^24^, 2006	Individuals with DS	127	2 to 24	Autism Behavior Checklist and Aberrant Behavior Checklist	Parents	DSM‐IV	-	2.33:1
Hepburn et al.^6^, 2007	Children with SD	20	2 to 3	Autism Diagnostic Interview-Revised and Autism Diagnostic Observation Schedule-Generic	Parents	DSM-IV TR	-	2.45:1
Dressler et al.^18^, 2011	Individuals with DS and/or autism living with their family	24	6 to 34	Childhood Autism Rating Scale (CARS)	Parents	DSM-IV TR	-	0.84:1
Ji et al.^25^, 2011	Individuals with DS	293	2 to 13	Autism Behavior Checklist	Not informed	DSM-IIIR and DSM-IV	-	3.16:1
Magyar et al.^28^, 2012	Individuals with DS	71	4 to 14	Archival Social Communication Questionnaire (SCQ), Autism Diagnostic Interview-Revised (ADI-R) and Autism Diagnostic Observation Schedule	Not informed	DSM-IV	-	1.29:1
Pandolfi et al.^7^, 2017	Individuals with DS	71	3 to 15	Pervasive Developmental Disorder in Mental Retardation Scale (PDD- MRS), Social Communication Questionnaire - Lifetime Version (SCQ-L) and Autism Diagnostic Interview-Revised (ADI-R)	Parents	DSM-IV- TR	-	1.15:1
Godfrey et al.^28^, 2019	Individuals with DS born between January 1, 1996, and December 21, 2003, and who had a caregiver who spoke English or Spanish fluently	33	born between 1996 and 2003	Autism Diagnostic Observation Schedule (ADOS) and Autism Diagnostic Interview-Revised (ADI-R)	Parents	DSM-V	-	2.01:00
Oxelgren et al.^29^, 2019	Individuals with DS	60	5 to 17	Autism Diagnostic Interview-Revised (ADI-R) and Autism Diagnostic Observation Schedule (ADOS)	Parents	Not informed	-	1.8:1
Capone et al.^29^, 2005	Individuals with DS	131	2 to 21	Behavior questionnaires, semi-structured neurological development assessment and observation during play or social interactions	Parents	DSM-IV	12.9%	2.7:1
DiGuiseppi et al.^8^, 2010	Individuals with DS born between January 1, 1996, and December 21, 2003, and who had a caregiver who spoke English or Spanish fluently	123	2 to 21	Behavior questionnaires, semi-structured neurological development assessment and observation during play or social interactions	Parents	DSM-IV TR	AUT 6.4% TEA 11.8% Total 18.2%	1.86:1
Moss et al.^30^, 2013	Participants were included if there was information on the date of birth and diagnosis of DS from a professional (physician, clinical geneticist, pediatrician or other), if at least 75% of the SCQ (Rutter et al., 2003) had been completed and if the participant with SD was at least 4 years of age.	108	4 to 62	Social Communication Questionnaire (SCQ)	Parents	Not informed	19%	0.7:1
Warner et al.^31^, 2014	Individuals with DS	160	4 to 40	Lifetime version of the Social Communication Questionnaire (SCQ) and Strengths and Difﬁculties Questionnaire	Parents	Not informed	AUT 16,5% TEA 37.7%	1.2:1

Regarding prevalence, there was variation in the results among the studies, as well as in the proportions of men and women in the sample composition. There was also heterogeneity among diagnostic outcomes, such as invasive developmental disorder (according to DSM-IV), ASD and autism, which were also related to the use of each diagnostic criterion.

Among the internationally validated scales for diagnosing ASD, eleven were used in the fifteen studies included in the systematic review. Seven of the tools used have a questionnaire format, for application to the children’s guardians (ADI-R, AutBC, ABC, SCQ, SCQ-L, PDD-MRS and SDQ), and the other four tools present the possibility of observation of the individual and interviewing the person responsible for the subject (ADOS, A-PL-ADOS, CARS and MCHAT). Among the tools used, four presented the diagnostic proposal (ADI-R, ADOS, A-PL-ADOS and CARS) and seven, the screening proposal (AutBC, ABC, SCQ, SCQ-L, PDD-MRS, M-CHAT and SDQ).

The minimum age at which subjects could be evaluated using these tools was 12 months, and two tools (A-PL-ADOS and PDD-MRS) were developed to evaluate individuals with cognitive impairments. There were differences in sensitivity and specificity, as presented in the table 4. It is important to highlight that sensitivity and specificity data may vary according to the study and the number of applications of the tool.

**Table 4.** General characteristics of the tools used for ASD evaluation^*^, ^**^, ^***^.

Tool	Description	Age	Sensitivity	Specificity	Amount of use (n = 15)
Autism Diagnostic Interview- Revised (ADI-R)^34^	[Diagnosis] Standardized questionnaire in accordance with DSM-IV criteria. This is an early childhood evaluation that checks social relationships, communication and repetitive behaviors.	from 18 months	0.52	0.84	8
Autism Diagnostic Observation Schedule (ADOS)^34^ ^35^	[Diagnosis] This is a semi-structured evaluation tool that enables observation of four areas: social interaction, communication, play and repetitive behaviors.	from 12 months	0.94	0.80	5
Adapted Pre-Linguistic Autism Diagnostic Observation Schedule (A-PL-ADOS)^36^	[Diagnosis] Tool developed for diagnosing autism that enables observation of children and adults with severe/profound cognitive impairments	from 3 years	0.82	0.85	1
Autism Behavior Checklist (AutBC)^37^	[Screening] Tool based on parents’ responses that evaluates behaviors associated with autism, in five subscales: sensory; interaction; body and object use; language; and social and self-help.	from 24 months	0.77	0.91	2
Aberrant Behavior Checklist (ABC)^38^	[Screening] This is a 58-item questionnaire that evaluates the severity of behaviors on five subscales: irritability; lethargy/social impairment; stereotyping; hyperactivity; and inadequate speech.	from 5 years	0.4-0.74	0.3-0.75	1
Childhood Autism Rating Scale (CARS)^34^	[Diagnosis] This is an tool for behavioral observations. It evaluates 15 items taking into account the symptoms for diagnosing ASD that are described in the DSM-IV	from 24 months	0.80	0.88	2
Archival Social Communication Questionnaire (SCQ)^390^	[Screening] This is a tool for evaluating individuals who are considered at risk of autism. It evaluates qualitative deficiencies in reciprocal social interaction and communication, as well as repetitive and stereotyped behavior	from 4 years	0.88	0.72	6
Social Communication Questionnaire - Lifetime Version (SCQ-L)^40^	[Screening] This version of the SCQ focuses on the child's development history, providing a total score that identifies individuals who may have autism and should be referred for a more thorough evaluation. The evaluation is practically unaffected by age, gender, language level and IQ performance	from 4 years	0.78	0.47	1
Pervasive Developmental Disorder in Mental Retardation Scale PDD- MRS^41^	[Screening] This is an ASD assessment tool that was developed for people with DS based on the DSM-IV-R criteria. It assesses the quality of social interactions with adults and colleagues, language and speech problems and aspects of behavior	from 2 years	0.92	0.92	1
Modified Checklist for Autism in Toddlers (M-CHAT)^42^	[Screening] This is a tool that is easy to apply and accessible, and it assesses psychometric properties. The caregiver responds to 23 yes/no items. Failure in three items or 2 out of 6 critical items (focus on joint attention, social orientation and imitation) indicates a risk of autism.	18 to 24 months	0.34	0.93	1
Strengths and Difﬁculties Questionnaire (SDQ)^43^	[Screening] This is an evaluation of 25 items that assesses the psychological condition of children and young people. It generates scores for emotional symptoms, behavioral problems, hyperactivity, peer problems and social behavior.	3 to 16 years	0.63	0.94	1

* Tests may vary according to more current versions, such as age references and values for specificity and sensitivity; ^**^Sensitivity and specificity data may vary according to studies and the way in which the tests are applied (single or double application); ^***^This table reports the ASD assessment tools contained in the studies included that are standardized.

## DISCUSSION

Several studies in this review evaluated identification of autism in the population with Down syndrome. It has been suggested in the existing literature that children with DS are different from those with DS and ASD^28^.

In the present study, 15 studies with poor quality of evidence, according to the criteria proposed by EPHPP (Effective Public Health Practice Project - Quality Assessment Toll for Quantitative Studies), were included. Their poor quality was mainly due to the selection bias and confounding factors present in them. In these studies, 11 different assessment tools were used, two of which are specifically directed to analysis of people with intellectual disabilities. No specific tool or scale for evaluating ASD in DS was found. The studies evaluated show that there is a need for greater dissemination of standardized scales for diagnosing ASD, since screening scales are not diagnostically definitive. It is also worth noting that in three studies it was not possible to identify the diagnostic criteria used.

Identification of early signs of autism risk has been widely studied in the general population, since the diagnosis tends to be made at the age of around three years. However, parents already report changes in the first year of life^44^.

Studies have reported that the stability of the ASD diagnosis in the general population reaches the rate of 75% around the age of three years. Identification of early signs of autism risk thus appears to provide a possibility for prevention and reduction of this disease^45^.

With the growing recognition through studies that a portion of the population with DS will present ASD in association with this, there is a need to identify early signs of risk and implement treatment as soon as these signs have been identified. This important for reducing the impacts of this comorbidity^7^.

Despite the persistent interest of the scientific community in this subject, only the study by Ortiz et. al^22^presented an outcome related to identification of early signs of autism risk in the population with DS.

In that retrospective study, the most significant early signs from the perspective of expert evaluators were identified through home videos of children who had already been diagnosed with autism. These evaluations were made through an instrument based on the Modified Checklist for Autism in Toddlers (M-CHAT) and the Autism Diagnostic Interview-Revised (ADI-R). The main findings were associated with absence of shared attention, reduced interest in other people, lack of eye contact, absence of imitation and the presence of repetitive and stereotyped movements. It was also pointed out in that study that, even with the difficulty of the DS population in processing stimuli, the clinician's watchful eye is needed in order to detect difficulties regarding shared attention and interest in social contact, and thus enable early diagnosis of ASD and effective intervention.

Screening tests are indicated for all children, including those with DS, since it is already possible to start intervention at an early age, for rehabilitation of children with a probable diagnosis of autism as a comorbidity^17^. One of the differential diagnoses of autism is intellectual disability, but it is noteworthy that both in children with DS and in those with ASD, the comorbidity of intellectual disability is also frequent, thus requiring assessment using specific cognitive scales. This differential diagnosis becomes more difficult as the cognitive impact increases^7^ ^24^ ^28^. Because of this complexity, it has been reported that the diagnosis of ASD in DS is made at older ages than in the general population^13^ ^16^ **.**

The diagnostic criteria most used as a reference, according to the studies included in the present review, were the DSM criteria. Most of the studies included in the present review used the DSM-IV and DSM-IV-TR as references, consequent to the years in which they were published ^6^ ^-^ ^8^ ^18^ ^24^ ^25^ ^27^ ^30^ ^32^. 

It is known that there is a relationship between intellectual disability and ASD and that, when the intellectual limitation is more significant, autism symptoms will be more evident. Thus, according to the DSM-V criteria, the individual must present a difference between these two impairments for there to be an additional diagnosis of ASD in cases of DS^33^.

In the ICD version 11, which is still in a preliminary version that is available on the WHO website, infantile autism and Asperger's syndrome are incorporated into ASD. Furthermore, categories have been created for this disorder, with and without intellectual and functional impairment^45^.

In the studies included in this review, wide variation in the prevalence of ASD in cases of DS was observed. All studies evaluating the prevalence of ASD in cases of DS found higher rates of the disorder than in the general population^8^ ^28^ ^30-32^. These studies also indicated that there was higher prevalence of ASD among men^8^ ^280^ ^30^ ^32^and among DS individuals with greater cognitive impairment. 

Several factors may influence the data on the prevalence of ASD in DS. The studies included point to possible influences relating to the diagnostic criterion used, sample recruitment method, socioeconomic aspects of the population studied, age, evaluation method (direct observation or interviews with parents or teachers), proportions between men and women and intellectual functioning. Because of all these different factors, it is not yet possible to specify the prevalence of the disorder in cases of DS. However, there is still consensus that the prevalence is higher than in the general population. This causes us to remain alert to occurrences of this comorbidity in the population with DS.

In this systematic review, the importance of applying a validated instrument for formal evaluation of ASD during the follow-up of children with DS was observed. One important point in choosing instruments is that screening tests show higher rates of false-positive ASD diagnoses in the population with DS^7^ ^8^ ^28^. This occurs because the screening instruments are affected by cognitive impacts and other conditions associated with DS^8^. 

It has been shown that the Social Communication Questionnaire (SCQ) has higher sensitivity and lower specificity rates, when used in the population with DS. However, performance data regarding ASD assessment tools remain limited among individuals with DS^7^ ^8^. 

In the studies included in this systematic review, the tools most used were the Autism Diagnostic Interview-Revised (ADI-Re) and the Autism Diagnostic Observation Schedule (ADOS), which are diagnostic tools that have not yet been validated in Brazil. Also used was the Archival Social Communication Questionnaire (SCQ), which is a screening tool already validated for use in Brazilian populations. When using these tools, it is important to verify the necessary adjustments for lower levels of intellectual functioning, as seen in cases of intellectual disability, whenever possible^24^.

Studies have indicated that screening tools such as M-CHAT R and SCQ should be used only for initial evaluations, since they are not sufficient to determine the diagnosis^24^ ^27^ ^31^. Even though it has been shown that the SCQ presents good convergence with gold standard tools, this application alone is not enough for the diagnosis. Thus, the diagnosis should be reached by also considering anamnesis, interviews, physical examinations, detailed observation and application of validated diagnostic scales^27^ ^31^. 

Star et al.^24^evaluated individuals with DS in association with severe or profound intellectual disability. The Autism Diagnostic Interview-Revised (ADI-R) and Adapted Pre-Linguistic Autism Diagnostic Observation Schedule (A-PL-ADOS) were used as tools. The latter is an instrument for evaluating nonverbal children and adults who have severe and profound intellectual limitations. In a sample of 13 individuals who had DS with serious intellectual impairments, five met the diagnostic criterion for autism. That study, despite its small sample, demonstrated that not all individuals who have serious intellectual impediments will be diagnosed with ASD.

Several studies have compared the profiles of individuals with ASD and DS, and those with DS only. These data show that individuals with ASD and DS have greater social withdrawal, aggressive behaviors and anxiety and worse social engagement than children with DS^7^ ^18^ ^25^ ^27^ ^28^ ^30^ ^31^. In addition, individuals with both diagnoses show lower levels of adaptive functioning^18^ and higher levels of repetitive and stereotyped behaviors, compared with those with DS alone^6^ ^25^ ^27^. 

At younger ages, when verbal communication skills are still developing, and especially in cases of DS (since delayed communication is expected in such cases), these characteristics will be more related to the qualitative aspects of communication (shared attention, interest, eye contact and imitation). Repetitive movements and stereotyping may also occur^23^.

In older children, when speech is present, more stereotyped and repetitive speech is expected. When speech is absent, limitation or absence of gesticulation with communicative objectives is observed. Greater aggressiveness in social contact, lack of symbolic and functional play, as well as a tendency to align objects and have restricted interests, can also be observed^7^ ^9^ ^25^. 

To make the diagnosis of ASD in DS, it is also necessary to consider the interference of factors associated with the syndrome. Sensory conditions, such as hearing loss and motor difficulties, for example hypotonia, can affect the time and fluidity of these individuals’ social and communicative behaviors. These signs are identified through screening methods, but differ qualitatively from the difficulty in basic social relationships seen in autism and may be misinterpreted if the examiner is not aware of the aspects relating to DS. Furthermore, it has been suggested that individuals with DS demonstrate executive function deficits that affect social and communicative relationships, but in a different way from the reciprocity problems associated with autism^8^.

Moreover, regarding the diagnosis of ASD, it is important to consider the conditions within which a differential diagnosis is necessary. Down Syndrome Disintegrative Disorder (DSDD) has been described as a clinical syndrome in which people with DS may experience adaptive, social and cognitive regression. Although DSDD may present symptoms similar to those of ASD, its onset is later, generally occurring between the first and third decades of life. Also, in DSDD there are other symptoms such as catatonia and insomnia. The differential diagnosis should be based on a comprehensive psychosocial and medical assessment of possible secondary causes of behavioral change and regression^46^.

Thus, to diagnose ASD in DS, clinicians should select appropriate tools, conduct analysis on intellectual development and functioning, make direct observations and conduct analysis on communication and social interaction and other aspects of social engagement, which are fundamental for distinguishing ASD from other developmental delays ^8^.

It is also worth mentioning the challenges faced by families with regard to the diagnosis of ASD. In a systematic review of the literature, it was observed that these challenges start with the search for a diagnosis, which may take a long time to be reached. There is the difficulty in dealing with the symptoms, and even in achieving access to rehabilitation, education and leisure services. These data emphasize the need to seek a systematic approach, starting from the time at which ASD is diagnosed, through appropriate care plans and support networks for children with ASD and their families^47^. These challenges are observed in the general population and may become greater in cases in which there is already a diagnosis of DS.

The present study had limitations with regard to the methodological and diagnostic system variations present in the 15 studies included, given that these factors interfere with identifying the best diagnostic practices. Future studies should use meta-analyses to address methodologies, in order to extract psychometric data from diagnostic practices in the population with DS.

Although we were unable to identify the most appropriate tool for evaluating ASD in DS, since the psychometric qualities of these tools are not well delimited for this population, this systematic review allowed us to understand that the clinical diagnosis of ASD in DS should not focus only on test results. Clinical experience and interdisciplinary evaluation will allow greater understanding of whether there is any qualitative difference in social engagement and cognitive impairment that would justify the second diagnosis of ASD in DS.

We highlight the need for early evaluation and intervention in cases of ASD associated with DS, since these will be determinants for better development, quality of life and social inclusion.

In conclusion, individuals with DS have higher prevalence of ASD than the general population, and screening should be universal, to enable early detection of signs and effective intervention, thus improving the prognosis in relation to the potential for development and better quality of life. The present systematic review showed that use of ASD diagnostic tools in the population with DS requires careful complementary and multidisciplinary clinical evaluation. In addition, there is a need to evaluate the psychometric properties of these tools in the population with DS, and whether tools that were created to evaluate people with intellectual disabilities present more affirmative results for the population with DS.

The need for additional diagnoses of ASD among individuals with DS should be determined based on the qualitative difference between social and cognitive impairments. It is also important to highlight the need to assess signs of autism risk in the first year of life, so that it becomes possible to analyze the qualitative aspects of social interaction and thus to initiate more timely intervention.

It is necessary to provide tools for early detection of autism risk among infants and for diagnostic evaluation of ASD in DS, based on developmental analyses in healthcare services, so that better results can be achieved with earlier interventions.

## References

[B1] Irving C, Basu A, Richmond S, Burn J, Wren C (2008). Twenty-year trends in prevalence and survival of Down syndrome. Eur J Hum Genet.

[B2] Rachubinski AL, Hepburn S, Elias ER, Gardiner K, Shaikh TH (2017). The co-occurrence of Down syndrome and autism spectrum disorder: is it because of additional genetic variations?. Prenat Diagn.

[B3] American Psychistric Association (2013). Diagnostic and Statistical Manual of Mental Disorders (DSM-V).

[B4] Maenner MJ, Shaw KA, Baio J, Washington A, Patrick M, DiRienzo M (2020). Prevalence of autism spectrum disorder among children aged 8 years - autism and developmental disabilities monitoring network, 11 sites, United States, 2016. MMWR Surveill Summ.

[B5] Sandin S, Lichtenstein P, Kuja-Halkola R, Larsson H, Hultman CM, Reichenberg A (2014). The familial risk of autism. JAMA.

[B6] Hepburn S, Philofsky A, Fidler DJ, Rogers S (2008). Autism symptoms in toddlers with Down syndrome: a descriptive study. J Appl Res Intellect Disabil.

[B7] Pandolfi V, Caroline IM, Charles A (2017). Screening for autism spectrum disorder in children with Down syndrome: An evaluation of the Pervasive Developmental Disorder in Mental Retardation Scale. J Intellect Dev Dis.

[B8] DiGuiseppi C, Hepburn S, Davis JM, Fidler DJ, Hartway S, Lee NR (2010). Screening for autism spectrum disorder in children with Down syndrome: An evaluation of the Pervasive Developmental Disorder in Mental Retardation Scale. J Dev Behav Pediatr.

[B9] Freeman NC, Gray KM, Taffe JR, Cornish KM (2016). A cross-syndrome evaluation of a new attention rating scale: the scale of attention in intellectual disability. Res Dev Disabil.

[B10] García-Primo P, Hellendoorn A, Charman T, Roeyers H, Dereu M, Roge B (2014). Screening for autism spectrum disorders: state of the art in Europe. Eur Child Adolesc Psychiatry.

[B11] Channell MM, Phillips BA, Loveall SJ, Conners FA, Bussanich PM, Klinger LG (2015). Patterns of autism spectrum symptomatology in individuals with Down syndrome without comorbid autism spectrum disorder. J Neurodev Disord.

[B12] Moher D, Liberati A, Tetzlaff J, Altman DG, PRISMA Group (2010). Preferred reporting items for systematic reviews and meta-analyses: the PRISMA statement. Int J Surg.

[B13] Jones EJH, Dawson G, Kelly J, Estes A, Webb SJ (2017). Parent-delivered early intervention in infants at risk for ASD: effects on electrophysiological and habituation measures of social attention. Autism Res.

[B14] Koegel L, Singh A, Koegel R, Hollingsworth J, Bradshaw J (2014). Assessing and improving early social engagement in infants. J Posit Behav Interv.

[B15] Laznik MC, Burnod Y, Szejer M, Kupfer MC (2016). Luzes sobre a clínica e o desenvolvimento de bebês: novas pesquisas, saberes e intervenções..

[B16] Berger JM, Rohn TT, Oxford JT (2013). Autism as the early closure of a neuroplastic critical period normally seen in adolescence. Biol Syst Open Access.

[B17] Pierce K, Gazestani VH, Bacon E, Barnes CC, Cha D, Nalabolu S (2019). Evaluation of the diagnostic stability of the early autism spectrum disorder phenotype in the general population starting at 12 months. JAMA Pediatr.

[B18] Parlato-Oliveira E Saint-Georges, Parlato-Oliveira E, Szejer M (2019). O Bebê e os desafios da cultura.

[B19] Olliac B, Crespin G, Laznik M-C, El Ganouni OCI, Sarradet J-L, Bauby C (2017). Infant and dyadic assessment in early community-based screening for autism spectrum disorder with the PREAUT grid. PLoS One.

[B20] Dressler A, Perelli V, Bozza M, Bargagna S (2011). The autistic phenotype in Down syndrome: differences in adaptive behaviour versus Down syndrome alone and autistic disorder alone. Funct Neurol.

[B21] Greenspan SI, Brazelton TB, Cordero J, Solomon R, Bauman ML, Robinson R (2008). Guidelines for early identification, screening, and clinical management of children with autism spectrum disorders. Pediatrics.

[B22] Effective Public Health Practice Project Hamilton R: quality assessment tool for quantitative studies.

[B23] Ouzzani M, Hammady H, Fedorowicz Z, Elmagarmid A (2016). Rayyan-a web and mobile app for systematic reviews. Syst Rev.

[B24] Ortiz B, Videla L, Gich I, Alcacer B, Torres D, Jover I, Videla S (2017). Early warning signs of autism spectrum disorder in people with Down syndrome. Int Med Rev Down Syndr.

[B25] Starr EM, Berument SK, Tomlins M, Papanikolaou K, Rutter M (2005). Brief report: autism in individuals with Down syndrome. J Autism Dev Disord.

[B26] Carter JC, Capone GT, Gray RM, Cox CS, Kaufmann WE (2007). Autistic-spectrum disorders in Down syndrome: further delineation and distinction from other behavioral abnormalities. Am J Med Genet B Neuropsychiatr Genet.

[B27] Ji NY, Capone GT, Kaufmann WE (2011). Autism spectrum disorder in Down syndrome: cluster analysis of aberrant behaviour checklist data supports diagnosis. J Intellect Disabil Res.

[B28] Magyar CI, Pandolfi V, Dill CA (2012). An initial evaluation of the Social Communication Questionnaire for the assessment of autism spectrum disorders in children with Down syndrome. J Dev Behav Pediatr.

[B29] Godfrey M, Hepburn S, Fidler DJ, Tapera T, Zhang F, Rosenberg CR (2019). Autism Spectrum Disorder (ASD) symptom profiles of children with comorbid Down Syndrome (DS) and ASD: a comparison with children with DS-only and ASD-only. Res Dev Disabil.

[B30] Oxelgren UW, Myrelid Å, Annerén G, Ekstam B, Göransson C, Holmbom A (2017). Prevalence of autism and attention-deficit-hyperactivity disorder in Down syndrome: a population-based study. Dev Med Child Neurol.

[B31] Capone GT, Grados MA, Kaufmann WE, Bernad-Ripoll S, Jewell A (2005). Down syndrome and comorbid autism-spectrum disorder: characterization using the aberrant behavior checklist. Am J Med Genet A.

[B32] Moss J, Richards C, Nelson L, Oliver C (2013). Prevalence of autism spectrum disorder symptomatology and related behavioural characteristics in individuals with Down syndrome. Autism.

[B33] Warner G, Moss J, Smith P, Howlin P (2014). Autism characteristics and behavioural disturbances in ~ 500 children with Down's syndrome in England and Wales. Autism Res.

[B34] Randall M, Egberts KJ, Samtani A, Scholten RJ, Hooft L, Livingstone N (2018). Diagnostic tests for Autism Spectrum Disorder (ASD) in preschool children. Cochrane Database Syst Rev.

[B35] Lombardo MV, Lai M-C, Baron-Cohen S (2019). Big data approaches to decomposing heterogeneity across the autism spectrum. Mol Psychiatry.

[B36] Lord C, Rutter M, Goode S, Heemsbergen J, Jordan H, Mawhood L (1989). Autism diagnostic observation schedule: a standardized observation of communicative and social behavior. J Autism Dev Disord.

[B37] Berument SK, Starr E, Pickles A, Tomlins M, Papanikolauou K, Lord C (2005). Pre-linguistic autism diagnostic observation schedule adapted for older individuals with severe to profound mental retardation: a pilot study. J Autism Dev Disord.

[B38] Eaves RC, Campbell HA, Chambers D (2000). Criterion‐Related and construct validity of the pervasive developmental disorders rating scale and the autism behavior checklist. Psychol Sch.

[B39] Rojahn J, Aman MG, Matson JL, Mayville E (2003). The Aberrant Behavior checklist and the behavior problems inventory: convergent and divergent validity. Res Dev Disabil.

[B40] Chandler S, Charman T, Baird G, Simonoff E, Loucas T, Meldrum D (2007). Validation of the social communication questionnaire in a population cohort of children with autism spectrum disorders. J Am Acad Child Adolesc Psychiatry.

[B41] Sappok T, Diefenbacher A, Gaul I, Bölte S (2015). Validity of the social communication questionnaire in adults with intellectual disabilities and suspected autism spectrum disorder. Am J Intellect Dev Disabil.

[B42] Kraijer D, Bildt A (2005). The PDD-MRS: an instrument for identification of autism spectrum disorders in persons with mental retardation. J Autism Dev Disord.

[B43] Stenberg N, Bresnahan M, Gunnes N, Hirtz D, Hornig M, Lie KK (2014). Identifying children with autism spectrum disorder at 18 months in a general population sample. Paediatr Perinat Epidemiol.

[B44] Cury CR, Golfeto JH (2003). Strengths and difficulties questionnaire (SDQ): a study of school children in Ribeirão Preto. Braz J Psychiatry.

[B45] Chawarska K, Klin A, Paul R, Volkmar F (2007). Autism spectrum disorder in the second year: stability and change in syndrome expression. J Child Psychol Psychiatry.

[B46] LaSalle JM (2013). Epigenomic strategies at the interface of genetic and environmental risk factors for autism. J Hum Genet.

[B47] Reed GM, First MB, Kogan CS, Hyman SE, Gureje O, Gaebel W (2019). Innovations and changes in the ICD-11 classification of mental, behavioural and neurodevelopmental disorders. World Psychiatry.

[B48] Rosso M, Fremion E, Santoro SL, Oreskovic NM, Chitnis T, Skotko BG (2020). Down syndrome disintegrative disorder: a clinical regression syndrome of increasing importance. Pediatrics.

[B49] Gomes PTM, Lima LHL, Bueno MKG, Araújo LA, Souza NM (2015). Autismo no Brasil, desafios familiares e estratégias de superação: revisão sistemática. J Pediatr (Rio J).

